# Perineural invasion as a risk factor for locoregional recurrence of invasive breast cancer

**DOI:** 10.1038/s41598-021-92343-4

**Published:** 2021-06-17

**Authors:** Priyanka Narayan, Jessica Flynn, Zhigang Zhang, Erin F. Gillespie, Boris Mueller, Amy J. Xu, John Cuaron, Beryl McCormick, Atif J. Khan, Oren Cahlon, Simon N. Powell, Hannah Wen, Lior Z. Braunstein

**Affiliations:** 1 Weill Cornell Medicine, New York, NY USA; 2 Department of Biostatistics and Epidemiology, Memorial Sloan Kettering Cancer Center, New York, USA; 3 Department of Radiation Oncology, Memorial Sloan Kettering Cancer Center, 1275 York Ave, Box 22, New York, NY 10044 USA; 4 Department of Pathology, Memorial Sloan Kettering Cancer Center, New York, USA

**Keywords:** Breast cancer, Cancer therapy

## Abstract

Perineural invasion (PNI) is a pathologic finding observed across a spectrum of solid tumors, typically with adverse prognostic implications. Little is known about how the presence of PNI influences locoregional recurrence (LRR) among breast cancers. We evaluated the association between PNI and LRR among an unselected, broadly representative cohort of breast cancer patients, and among a propensity-score matched cohort. We ascertained breast cancer patients seen at our institution from 2008 to 2019 for whom PNI status and salient clinicopathologic features were available. Fine-Gray regression models were constructed to evaluate the association between PNI and LRR, accounting for age, tumor size, nodal involvement, estrogen receptor (ER), progesterone receptor (PR), HER2 status, histologic tumor grade, presence of lymphovascular invasion (LVI), and receipt of chemotherapy and/or radiation. Analyses were then refined by comparing PNI-positive patients to a PNI-negative cohort defined by propensity score matching. Among 8864 invasive breast cancers, 1384 (15.6%) were noted to harbor PNI. At a median follow-up of 6.3 years, 428 locoregional recurrence events were observed yielding a 7-year LRR of 7.1% (95% CI 5.5–9.1) for those with PNI and 4.7% (95% CI 4.2–5.3; p = 0.01) for those without. On univariate analysis throughout the entire cohort, presence of PNI was significantly associated with an increased risk of LRR (HR 1.39, 95% CI 1.08–1.78, p < 0.01). Accounting for differences in salient clinicopathologic and treatment parameters by multivariable Fine-Gray regression modeling, the association between PNI and LRR was potentiated (HR 1.57, 95% CI 1.2–2.07, p = 0.001). We further conducted propensity score matching to balance clinicopathologic parameters and treatments between the two groups (PNI vs not), again showing a similar significant association between PNI and LRR (HR 1.46, 95% CI 1.03–2.08, p = 0.034). PNI is significantly associated with LRR following the definitive treatment of invasive breast cancer. The excess risk conferred by PNI is similar in magnitude to that observed with LVI, or by ER/PR negativity. Breast cancer prognostication and therapeutic decision-making should consider the presence of PNI among other salient risk factors. Larger studies among more uniform breast cancer presentations may elucidate the extent to which these findings apply across breast cancer subtypes and stages.

## Introduction

Breast cancer is the most common non-cutaneous malignancy in the United States, affecting up to 1 in 8 women by the age of 70^1^. While surgical resection is universally employed in the curative management of breast cancer, the selection of adjuvant therapies, such as chemotherapy or radiation, depends largely on the underlying clinicopathologic features of the tumor^2–7^.

Perineural invasion (PNI) is a well-described pathologic finding seen among various malignancies, including those of the breast^8^. Whereas lymphatic and vascular modes of cellular spread from a primary tumor are understood to be the main avenues of metastatic dissemination, PNI is less well studied, yet has been identified for over a century among pathologic specimens. Moreover, whereas PNI is strongly associated with adverse outcomes among several malignancies^9–12^, a small body of breast cancer literature is mixed in terms of identifying an association between PNI and breast cancer outcomes, leading to uncertainty about the consideration of PNI in adjuvant treatment selection^13–15^.

Different growth patterns have been variably described as PNI^8^. These have included a breadth of definitions spanning tumor within any layer of the peripheral nerve sheath, to distinct clusters of cancer cells adjacent to a nerve that is otherwise surrounded by normal tissue. A seminal 1985 paper broadly defined PNI as comprising tumor “in, around, and through the nerves”, although this definition has subsequently been refined^16^. For the purposes of this study, PNI was defined as invasion of tumor cells into any of the perineural compartments—a definition that is now used routinely in clinical pathology practice.

In this report, we identified breast cancer patients with broadly-defined PNI in an effort to evaluate locoregional outcomes in comparison to unselected and matched cohorts. We sought to determine the extent to which this pathologic finding is associated with breast cancer recurrence, potentially affording an opportunity for risk-adapted treatment.

## Methods

### Study population

We identified evaluable breast cancer patients presenting to our institution from 2008 to 2019 for whom comprehensive pathologic and treatment data were available. Clinicopathologic features were collected including patient age, tumor size, number of involved lymph nodes, estrogen receptor (ER), progesterone receptor (PR), and HER2 status, tumor histologic grade and lymphovascular invasion. PNI was universally reported as present or absent on pathologic evaluation at our center starting in 2018, although cases from prior to this date were ascertained if PNI was specifically noted (n = 31 patients from 2018 onwards in this analysis). Treatment parameters were also collected, including surgery type (mastectomy or partial mastectomy), margin status, and whether patients received chemotherapy, hormonal therapy, or radiation. This study was approved by the Memorial Sloan Kettering Institutional Review Board and the requirement for informed consent was waived due to the aggregated retrospective nature of the analyses. All methods adhered to HIPAA rules and were performed in accordance with the Declaration of Helsinki guidelines.

### Statistical analysis

The primary outcome of interest was locoregional recurrence (LRR), defined as the time from surgery to first recurrence in the ipsilateral breast and/or lymph nodes, with death as a competing risk. If the patient had multiple re-excisions, the time from last surgery was used. Patient and treatment characteristics were summarized using median and range for continuous variables and counts for categorical variables. Histopathologic characteristics were compared using the Chi-squared test for categorical variables, and Wilcoxon rank sum test for continuous variables. A multivariable Fine-Gray regression model was then constructed using clinically relevant covariates. Analyses were conducted on both the overall cohort (those with PNI versus those without) and on a matched cohort (developed utilizing propensity score matching for traits among those with PNI to traits among those without) to ensure similar features between the two groups (i.e. age, tumor size, nodal involvement, ER, PR, HER2, grade, LVI, surgical approach, laterality, chemotherapy and radiation). All statistical analyses were conducted with a type I error rate (α) of 0.05 and were performed using R version 3.6.2 (R Core Development Team, Vienna, Austria).

## Results

### Patient characteristics

We identified 8864 invasive breast cancer patients treated definitively at our center from 2008 to 2019 for whom complete relevant clinicopathologic data were available (Table 1). The cohort included 1384 patients whose primary tumor harbored PNI and 7480 for whom PNI was not reported. Among those harboring PNI, the median age was 57 (range 23–95), with a median tumor size of 1.9 cm and a median of no involved regional lymph nodes. Chemotherapy was administered to 65% of those with PNI and radiation to 68%, in contrast to 73% and 42%, respectively, among those not definitively exhibiting PNI. At a median follow-up of 6.3 years for the overall cohort (6.5 years for those without PNI; 5.1 years for those with PNI), 428 locoregional recurrence events were observed.

**Table 1:** Patient and treatment characteristics.

Perineural invasion (PNI)	PNI absent, N = 7480	PNI present, N = 1384
Age	52 (36, 69)	57 (23, 95)
Tumor size (cm)	1.50 (0.00, 18.50)	1.90 (0.00, 16.00)
Lymph nodes involved	0.00 (0.00, 53.00)	0.00 (0.00, 51.00)
**ER**		
Negative	1627 (22%)	116 (8.4%)
Positive	5853 (78%)	1268 (92%)
**PR**		
Negative	2723 (36%)	214 (15%)
Positive	4757 (64%)	1170 (85%)
**HER2**		
Negative	6184 (83%)	1268 (92%)
Positive	1296 (17%)	116 (8.4%)
**Histologic grade**		
1	493 (6.6%)	34 (2.5%)
2	2101 (28%)	266 (19%)
3	4886 (65%)	1084 (78%)
**LVI**		
Negative	4826 (65%)	634 (46%)
Positive	2654 (35%)	750 (54%)
**Surgery**		
Mastectomy	3543 (47%)	565 (41%)
Partial mastectomy	3937 (53%)	819 (59%)
**Side**		
Left	3743 (50%)	704 (51%)
Right	3737 (50%)	680 (49%)
**Margins**		
Close, (< 2 mm)	265 (3.6%)	34 (5.3%)
Negative	6578 (90%)	581 (90%)
Positive	437 (6.0%)	31 (4.8%)
Unknown	200	738
Chemotherapy administered	5475 (73%)	899 (65%)
Radiation administered	3126 (42%)	946 (68%)

Statistics presented: median (range); n (%).

### Univariate and multivariable analyses of the association between PNI and locoregional recurrence among the overall cohort

Univariate analysis of clinicopathologic and treatment characteristics revealed several well-established associations with LRR (Table 2): Increasing age was associated with lower risk of LRR (HR 0.98 per year, 95% CI 0.97–0.99, p = 0.001), as were ER or PR positivity (HR 0.55 and 0.69, respectively, p < 0.001 for each), while presence of LVI was associated with increased LRR (HR 1.33, 95% CI 1.1–1.61, p = 0.003). Notably, presence of PNI was significantly associated with an increased risk of LRR (HR 1.39, 95% CI 1.08–1.78, p < 0.01).

**Table 2:** Univariate analysis of locoregional recurrence by clinicopathologic features throughout the overall cohort.

Characteristic	HR (95% CI)	p-value
Presence of PNI	1.39 (1.08–1.78)	0.01
Age (per year)	0.98 (0.97–0.99)	0.001
Tumor size (per cm)	1.05 (1–1.1)	0.073
Lymph nodes involved (per node)	1 (0.98–1.02)	0.92
ER-negative	1.83 (1.49–2.24)	< 0.001
PR-negative	1.44 (1.19–1.75)	< 0.001
HER2+	1.04 (0.81–1.34)	0.754
High histologic grade	1.45 (1.16–1.8)	0.001
Lymphovascular invasion	1.33 (1.1–1.61)	0.003
Lumpectomy (vs mastectomy)	0.39 (0.32–0.48)	< 0.001
Laterality (right vs left)	0.87 (0.72–1.05)	0.14
**Margins (vs negative)**		0.003
< 2 mm	1.73 (1.11–2.68)	
Positive	1.61 (1.11–2.33)	
Chemotherapy administered	1.36 (1.07–1.74)	0.014
Radiotherapy administered	1.17 (0.97–1.42)	0.098

Given the various differences between patients harboring PNI and those without, a multivariable Fine-Gray regression model was constructed to elucidate the association between PNI and LRR, controlling for the other clinicopathologic and treatment features (Table 3). In this model, accounting for age, tumor size, nodal status, ER, PR, HER2, grade, LVI, chemotherapy and radiation, the presence of PNI remained significantly associated with LRR and the effect size was potentiated beyond that seen on univariate analysis (HR 1.57, 95% CI 1.2–2.07, p = 0.001).

**Table 3:** Multivariable analysis of locoregional recurrence by clinicopathologic features.

Characteristic	HR (95% CI)	p-value
Presence of PNI	1.57 (1.2–2.07)	0.001
Age (per year)	0.98 (0.97–0.99)	> 0.001
Tumor size (per cm)	1 (0.94–1.07)	0.99
Lymph nodes involved (per node)	0.98 (0.96–1.01)	0.21
ER-negative	1.75 (1.3–2.34)	< 0.001
PR-negative	1.12 (0.85–1.47)	0.42
HER2+	0.87 (0.67–1.13)	0.29
High histologic grade	1.15 (0.91–1.46)	0.24
Lymphovascular invasion	1.24 (1–1.54)	0.054
Chemotherapy administered	1 (0.75–1.34)	0.99
Radiotherapy administered	1.13 (0.92–1.39)	0.24

### Univariate and multivariate analyses of the association between PNI and locoregional recurrence in a propensity matched cohort

In a further effort to account for the observed imbalance in salient features between PNI positive and negative patients, propensity score matching was conducted to balance clinicopathologic parameters and treatments between the two groups (Table 4). All 1320 PNI positive patients were matched to 1320 PNI negative patients on salient features including age, tumor size, lymph node involvement, ER, PR, HER2, grade, LVI, surgery, side, chemotherapy, and radiation. A slight residual imbalance remained between the groups with regard to tumor size (with PNI positive patients having a median 2 mm larger tumor).

**Table 4:** Clinicopathologic characteristics of patients with PNI and matched PNI-negative controls.

Characteristic	0, N = 1320^a^	1, N = 1320^a^	p-value^b^
Age	58 (50, 64)	56 (48, 65)	0.6
Tumor size	1.70 (1.10, 2.50)	1.90 (1.30, 2.60)	< 0.001
LNs	0.0 (0.0, 2.0)	0.0 (0.0, 2.0)	0.6
**ER**			0.5
Positive	1195 (91%)	1205 (91%)	
Negative	125 (9.5%)	115 (8.7%)	
**PR**			0.8
Positive	1101 (83%)	1106 (84%)	
Negative	219 (17%)	214 (16%)	
**HER2**			0.9
Negative	1202 (91%)	1205 (91%)	
Positive	118 (8.9%)	115 (8.7%)	
**Histologic grade**			0.4
1–2	281 (21%)	299 (23%)	
3	1039 (79%)	1021 (77%)	
**LVI**			0.4
Negative	647 (49%)	626 (47%)	
Positive	673 (51%)	694 (53%)	
**Surgery**			0.3
Mastectomy	576 (44%)	546 (41%)	
Partial mastectomy	744 (56%)	774 (59%)	
**Side**			> 0.9
Left	667 (51%)	664 (50%)	
Right	653 (49%)	656 (50%)	
Chemo	897 (68%)	877 (66%)	0.4
Radiation	873 (66%)	886 (67%)	0.6

^a^Statistics presented: median (IQR); n (%).

^b^Statistical tests performed: Wilcoxon rank-sum test; chi-square test of independence.

On univariate analysis between these matched cohorts, PNI remained significantly associated with LRR (HR 1.46, 95% CI 1.03–2.06, p = 0.034). Multivariable analysis was similarly undertaken to account for salient and modestly imbalanced clinicopathologic features including age and tumor size. Adjusting for these variables, the association between PNI and LRR remained unchanged (HR 1.46, 95% CI 1.03–2.08, p = 0.034) suggesting that little of the observed association in the univariate model was due to residual confounding by imbalances in the groups (Table 5).

**Table 5:** Locoregional recurrence among those with PNI compared to a PNI-negative propensity-matched cohort.

	HR (95% CI)	p-value
**Univariate characteristic**		
Presence of PNI	1.46 (1.03–2.06)	0.034
**Multivariable model (among unbalanced covariates)**		
Presence of PNI	1.46 (1.03–2.07)	0.034
Age (per year)	0.98 (0.97–1)	0.088
Tumor size (per cm)	1.02 (0.95–1.09)	0.64
Lymph nodes involved (per node)	1.02 (0.98–1.06)	0.31

### Locoregional recurrence rates

**Figure 1:**
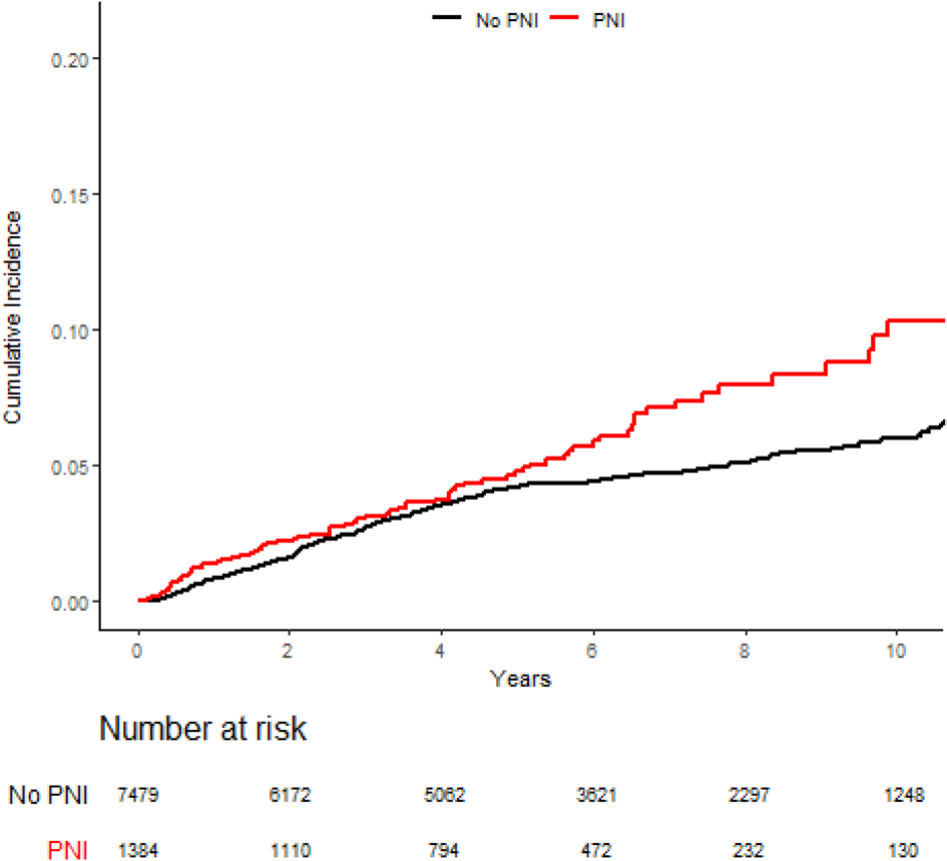
Locoregional recurrence by PNI status over the entire cohort.

**Figure 2:**
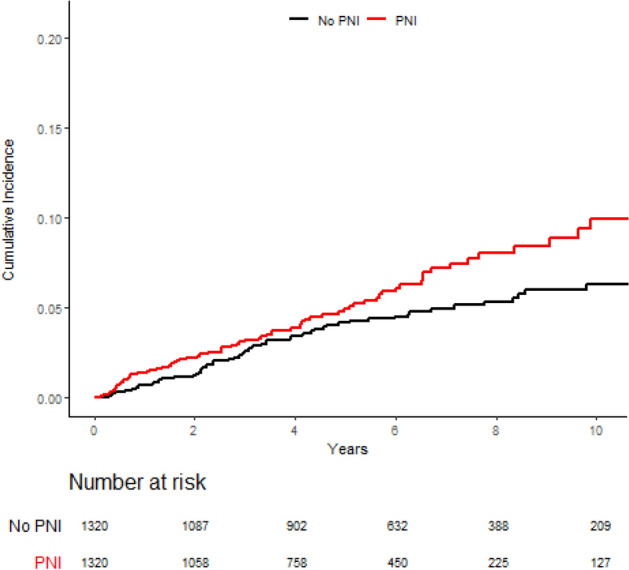
Locoregional recurrence among those with PNI compared to a cohort of PNI-negative propensity-matched controls.

With a median follow-up of 6.3 years for the overall cohort, the 7-year rate of LRR differed significantly between those with PNI (7.1%; 95% CI 5.5–9.1) and those without PNI (4.7%; 95% CI 4.2–5.3; p = 0.01) (Fig. 1). A similar comparison by PNI status among propensity-matched patients revealed a similarly significant difference in 7-year LRR between those with PNI (7.2%; 95% CI 5.5–9.2), and those without (5.0%; 95% CI 3.7–6.4; p = 0.034) (Fig. 2).

## Discussion

These analyses demonstrate that PNI represents a significant risk factor associated with LRR following the definitive treatment of invasive breast cancer. This association was observed on both univariate and multivariate analyses of the overall unselected cohort, and on analyses following propensity score matching, with an excess risk of LRR attributable to PNI approximating 40–60%. Whereas PNI has been described as an adverse risk factor among other malignancies, its implications for breast cancer have hitherto remained unclear. This report represents the largest analysis to date of PNI in invasive breast cancer, characterizing it as an adverse risk factor for LRR.

Several prior studies have evaluated the role of PNI with regard to LRR, albeit among smaller, now-outdated cohorts, and often in conjunction with other pathologic findings. Mate et al., for example, evaluated a cohort of 188 women with early stage breast cancer, concluding in 1986 that PNI did not affect recurrence outcomes, although neither did LVI or tumor grade^17^. Roses et al., conversely, concluded that only LVI (again, not PNI) affected recurrence in a similar cohort of 122 T1N0 patients^18^. Meanwhile, McCready et al. undertook two retrospective reviews, both suggesting that LVI and PNI, consolidated into a single risk factor, both were associated with recurrence^19,20^.

The literature has been similarly mixed in subsequent years, with Duraker et al. finding that PNI was more likely to be found in hormone-sensitive, mixed type, or ductal carcinoma and less likely to be found in axilla-negative or smaller tumors, however, without implications for recurrence^15^. With regard to other associated risk factors, PNI has been found predict for the involvement of > 3 lymph nodes^21^. Similarly, a 2010 single institution study of 1136 cases (of which only 13 had PNI) found that PNI was associated with LVI and larger tumors, although the paucity of PNI-bearing cases limited outcome analyses^13^.

Our findings must be interpreted in the context of the study design. By virtue of the retrospective nature of these analyses, the underlying results regarding PNI are subject to potential confounding by other risk factors for locoregional recurrence. That is, PNI is itself associated with several known risk factors, as seen in Table 1. We attempted to account for this clinicopathologic correlation by conducting both multivariable analyses and propensity score matching, both of which yielded consistent findings of similar magnitude with regard to the contribution of PNI to LRR. Moreover, as with any retrospective study in which therapeutic decisions are not held constant, our study may have been subject to confounding by indication whereby clinicians were influenced to select particular therapies by the finding of PNI. As above, multivariable models that included surgical approach, chemotherapy, endocrine therapy and radiotherapy were used to control for the influence of treatment selection and, moreover, analysis by propensity score matching permitted comparison of largely similar cohorts. An additional consideration is one that arises often throughout the breast cancer literature with regard to studying LRR as a composite endpoint for local and regional recurrences. Given the relative paucity of regional recurrences for which no comprehensive surveillance testing exists, the reliability of detecting nodal recurrences before distant metastases arise is uncertain. Moreover, the profound influence of surgical approach (mastectomy versus breast conservation) on patterns of recurrence can also obfuscate associations between clinicopathologic features and recurrence types. Thus, we were unable to robustly deduce whether PNI itself is associated more with local versus regional recurrences, or whether therapeutic selection was a more prominent factor in the recurrence patterns we observed.

Here, we suggest that PNI may be a relevant and significant risk factor for LRR among patients with definitively-treated breast cancer. As with other adverse clinicopathologic features, such as young age, lymphovascular invasion, high grade, etc., adjuvant therapy selection should take into account the entire risk landscape in tailoring therapeutic benefit for a given patient. The promise of precision medicine should ultimately incorporate the breadth of these features into an individualized risk estimate for each patient.
